# Slot Antenna Integrated Re-Entrant Resonator Based Wireless Pressure Sensor for High-Temperature Applications

**DOI:** 10.3390/s17091963

**Published:** 2017-08-25

**Authors:** Shujing Su, Fei Lu, Guozhu Wu, Dezhi Wu, Qiulin Tan, Helei Dong, Jijun Xiong

**Affiliations:** 1 Key Laboratory of Instrumentation Science & Dynamic Measurement, Ministry of Education, North University of China, Taiyuan 030051, China; sushujing@nuc.edu.cn (S.S.); lufei_55@163.com (F.L.); pillar921@163.com (G.W.); donghelei@nuc.edu.cn (H.D.); xiongjijun@nuc.edu.cn (J.X.); 2 Science and Technology on Electronic Test and Measurement Laboratory, North University of China, Taiyuan 030051, China; 3 Department of Mechanical & Electrical Engineering, Xiamen University, Xiamen 361005, China; wdz@xmu.edu.cn

**Keywords:** re-entrant resonator, slot antenna, wireless pressure sensor, high temperature, background clutter compensation

## Abstract

The highly sensitive pressure sensor presented in this paper aims at wireless passive sensing in a high temperature environment by using microwave backscattering technology. The structure of the re-entrant resonator was analyzed and optimized using theoretical calculation, software simulation, and its equivalent lump circuit model was first modified by us. Micro-machining and high-temperature co-fired ceramic (HTCC) process technologies were applied to fabricate the sensor, solving the common problem of cavity sealing during the air pressure loading test. In addition, to prevent the response signal from being immersed in the strong background clutter of the hermetic metal chamber, which makes its detection difficult, we proposed two key techniques to improve the signal to noise ratio: the suppression of strong background clutter and the detection of the weak backscattered signal of the sensor. The pressure sensor demonstrated in this paper works well for gas pressure loading between 40 and 120 kPa in a temperature range of 24 °C to 800 °C. The experimental results show that the sensor resonant frequency lies at 2.1065 GHz, with a maximum pressure sensitivity of 73.125 kHz/kPa.

## 1. Introduction

Pressure measurement in harsh environment, e.g., an internal combustion engine chamber, high-temperature surrounding, or a biological body, is a crucial problem that urgently requires a solution. The precise measurement of the pressure at key parts of the equipment of a system can provide in situ data on its mechanism, thus building a foundation for safe operation and improved efficiency, thereby attracting the efforts of many researchers towards its realization. As per previous reports, the pressure sensor based on the piezoresistive effect is affected by the inevitable P–N junction leakage current at elevated temperatures, which causes failure at high operating temperatures [1,2], while the optical-based sensor requires a high surface roughness of the membrane and reliable sealing of several key connections [3,4]. In addition, the sensor based on wired connections fails to work in some extreme conditions, e.g., on rotating turbine blades. As a result, studies on wireless sensors with passive working mechanisms were conducted later. For example, the surface acoustic wave (SAW) sensor could work up to 900 °C with a sensitivity of 3.3 kHz/kPa [5,6]. However, its sensor signal is easily contaminated by the environment, and the instability of the substrate material under high-temperature conditions restricts its operation. In present times, the wireless passive LC resonant sensor seems to be the most promising option. While they can work up to 800 °C using high-temperature co-fired ceramic (HTCC) technique [7,8] displaying a maximum sensitivity of 7.9 kHz/kPa, their quality (*Q*) factor deteriorates quickly at elevated temperatures. Thus, the electromagnetically coupled signal gets absorbed by the metal ground, which renders the response signal undetectable near the surface of the metal.

On account of the above considerations and owing to the advantages of enhanced sensitivity, substantially reduced volume and weight, and possibility of monolithic integration, devices which combine high *Q* factor resonators with microwave backscattering technology have increasingly attracted our interest [9,10,11]. Among them, evanescent-mode waveguide filters have the advantages of low loss, high selectivity, and tunable filtering along with reconfigurable RF front-ends [12,13]. It is well known that waveguides being cut off can be designed as microwave filters by adding a conductive re-entrant post inside them. This is equivalent to the introduction of an effective shunt capacitance. By changing the capacitance, i.e., the gap between the end post and the waveguide wall, the central frequency of the filter can be changed.

Based on previous studies, a harsh environment oriented, pressure-modulating re-entrant resonator sensor, constructed using alumina ceramic has been proposed in this paper for wireless passive pressure measurement. It displays a high sensitivity of 73.125 kHz/kPa which is approximately 21-fold and 9-fold higher than the sensitivities of the SAW sensor [6] and the LC sensor [9], respectively. An application of the above principle is found in [11] for the design of a pressure sensor based on a cavity resonator structure that works in atmospheric environment, in which the pressure is loaded through a dielectric rod. This technique of pressure loading is not suitable in a harsh environment. 

Moreover, the pressure sensor proposed in this work has the advantages of no additional sealing and packing requirements for gas pressure measurement. However, the measurement being conducted inside a hermetical metal chamber poses great challenges. Thereby, we propose two methods to solve the problem of difficulty in signal detection in a metallic environment and realize pressure measurement from 40 to 120 kPa, corresponding to a temperature range between 24 °C and 800 °C.

## 2. Principle of Measurement and Design of the Sensor

**Figure 1:**
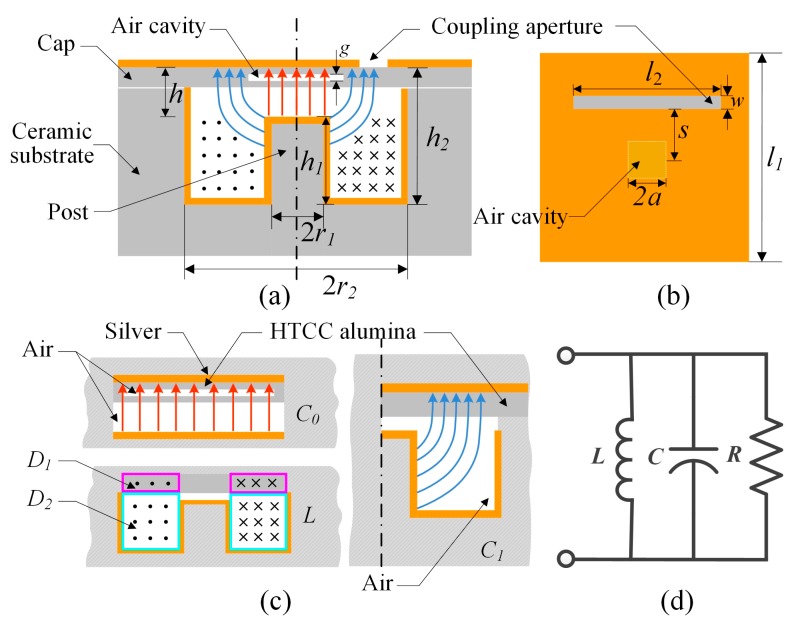
Model of the proposed re-entrant resonator pressure sensor. (**a**) Cross-sectional view of the re-entrant resonator; (**b**) Top view of the cavity cap; (**c**) Illustration of the equivalent capacitors *C*_0_, *C*_1_, and the inductance *L*; (**d**) Equivalent lumped circuit model of the re-entrant resonator structure.

The structure of the proposed cylindrical re-entrant resonator substrate integrated with a rectangular cap is illustrated in Figure 1a,b. Here, the re-entrant resonator is selected as the pressure-sensitive structure owing to its closed metal cavity, which results in a relatively high *Q* factor, high sensitivity, and a smaller dimension compared to the lumped LC sensor at the same operation frequency. Because the dimension of the re-entrant resonator is smaller than the resonant wavelength, and the cavity gap *h* is smaller than its height *h*_2_, the electric and the magnetic fields corresponding to the dominant mode of the resonant cavity are concentrated in the cylindrical volume of gap *h* and the bottom annular domain of the post with height *h*_1_, respectively. This is depicted in Figure 1a. The lumped circuit model can be used to calculate the capacitance *C*, inductance *L,* and resistance *R* of the re-entrant resonator.

According to [14], the equivalent capacitance *C* of the re-entrant resonator includes two parts, *C*_0_ and *C*_1_, where, *C*_0_ is the parallel capacitance between the post top and the central cap, i.e., the red section in Figure 1a, and *C*_1_ is the capacitance between post side wall and the end cap, i.e., the blue section in Figure 1a. This is in good agreement with the simulation result which was obtained using the ANSYS HFSS software (ANSYS, Inc., Canonsburg, PA, USA). The capacitance *C*_0_ shown in Figure 1c, can be represented by two parallel-plate capacitors in series containing different dielectric media i.e., alumina and air. We can calculate *C*_0_ as

(1) $$C_{0}=\frac{\varepsilon_{0}\pi r_{1}^{2}}{h-2g+\frac{2g}{\varepsilon_{r}}}$$

where *ε*_0_ denotes relative permittivity of vacuum, i.e., 8.854 × 10^−12^ F/m. *ε_r_* denotes relative permittivity of alumina.

Furthermore, *C*_1_ is viewed as a perpendicular-plate capacitor containing alumina and air dielectrics, as shown in Figure 1c. For the convenience of brief calculation, we approximate it as two series capacitors, where one is a parallel-plate capacitor filled with dielectric alumina, and the other is a perpendicular-plate capacitor filled with dielectric air. Hence, *C*_1_ can be calculated as

(2) $$C_{1}={\left({\left[4\varepsilon_{0}r_{1}\ln\frac{e\sqrt{{\left(h_{2}-3g\right)}^{2}+{\left(r_{2}-r_{1}\right)}^{2}}}{2\left(h-3g\right)}\right]}^{-1}+{\left[\frac{\varepsilon_{0}\varepsilon_{r}\pi \left(r_{2}{}^{2}-r_{1}{}^{2}\right)}{3g}\right]}^{-1}\right)}^{-1}$$

where *g* is the thickness of a single layer of HTCC prepared alumina, which is 0.1 mm for the one used in the present work. Because of the rectangular shape of the air cavity, here we neglect the capacitance effect caused by the four corners of the air cavity and the side wall of the post for its weak effect. The equivalent capacitance *C* of the re-entrant resonator sensor model can be expressed as

(3) $$C=C_{0}+C_{1}$$

The equivalent inductance *L* of the model can be regarded as two inductors in series, having annular coils with one turn each. As shown in Figure 1c, the cross-sectional diameters of the annular inductors with air and alumina as dielectrics are *D*_1_ and *D*_2_*,* respectively. By modifying the expression in [14], the final equivalent inductance *L* can be calculated as

(4) $$L=\frac{\mu_{0}\left(h_{2}-3g\right)}{2\pi }\ln\frac{r_{2}}{r_{1}}+\frac{\mu_{0}\mu_{r}3g}{2\pi }\ln\frac{r_{2}}{r_{1}}$$

In the Expression (4), the first and the second term denote the annular inductance for air, and alumina respectively. *μ*_0_ represents the relative permeability of vacuum, with a value 4*π* × 10^−7^ H/m and *μ_r_* is the relative permeability of alumina. Here, to simplify the analysis, the air in the re-entrant resonator and the HTCC prepared alumina cap are considered to be non-magnetic materials with a relative permeability of 1. Hence, the equivalent inductance can be simplified as

(5) $$L=\frac{\mu_{0}h_{2}}{2\pi }\ln\frac{r_{2}}{r_{1}}$$

The resonant frequency of an equivalent shunt lumped circuit model can be expressed as

(6) $$f_{0}=\frac{1}{2\pi \sqrt{LC}}$$

By substituting Expressions (3) and (5) for *C* and *L*, Expression (6) can be calculated as

(7) $$\begin{array}{l} f_{0}={\left(2\pi \mu_{0}h_{2}\ln\frac{r_{2}}{r_{1}}\right)}^{-\frac{1}{2}}\cdot {\left\{\varepsilon_{0}\pi r_{1}{}^{2}/\left[\left(h-2g\right)+2g/\varepsilon_{r}\right]+\frac{M\cdot N}{M+N}\right\}}^{-\frac{1}{2}}\\ \left(M=4\varepsilon_{0}r_{1}\ln\frac{e\sqrt{{\left(h_{2}-3g\right)}^{2}+{\left(r_{2}-r_{1}\right)}^{2}}}{2\left(h-3g\right)},N=\frac{\varepsilon_{0}\varepsilon_{r}\pi \left(r_{2}{}^{2}-r_{1}{}^{2}\right)}{3g}\right) \end{array}$$

**Figure 2:**
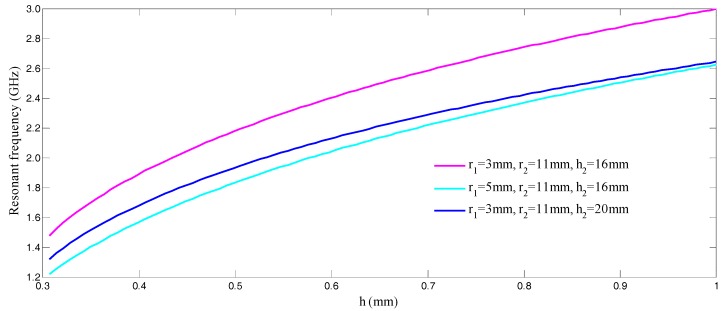
Re-entrant resonator resonant frequency variation with gap *h* (*h* > 0.3 mm).

Since there are totally 11 variables for determination of the sensor dimension as showed in Expressions (3)–(7), MATLAB software is used via initialization of variables to qualitatively study the variation of the sensor resonant frequency as a function of increasing pressure. Figure 2 illustrates the change in resonant frequency of three different cavity configurations with a variation of *h*. Here, we use *h* to characterize the amount of loaded pressure. For the sensor designed by us, the re-entrant resonator cap depicted in Figure 1a, with a sealed cavity is viewed as the pressure sensitive part. When the gas pressure loaded on the sensor increases, the top and bottom films of the cap deform inwards, towards the sealed HTCC cavity. It can be seen from Figure 2 that with the decrease of the gap *h*, the theoretical resonant frequency *f*_0_ of the re-entrant resonator decreases and the sensitivity of the sensor increases. A comparison of three cavity configurations demonstrates that the smaller the inner radius *r*_1_ and height *h*_2_, the higher the sensitivity of the sensor. For the dimension designed for this sensor, according to [11,15], the *Q* factor of the re-entrant resonator will increase with an increase in the gap *h*, thus causing an increase in the sensing distance. Besides, a larger dimension of the sensor will lead to a smaller resonant frequency, resulting in reduced propagation path loss, leading to increase in the sensing distance. Taking into consideration both the sensitivity and sensing distance of the sensor, a tradeoff should be made between its sensitivity Δ*f*_0_/Δ*h* and *Q* factor. Thus, the dimension (*r*_1_ = 3 mm, *r*_2_ = 11 mm, *h* = 0.7 mm, *h*_2_ = 16 mm) which has been preliminarily chosen for our design denotes a cavity resonant frequency of 2.58 GHz.

**Figure 3:**
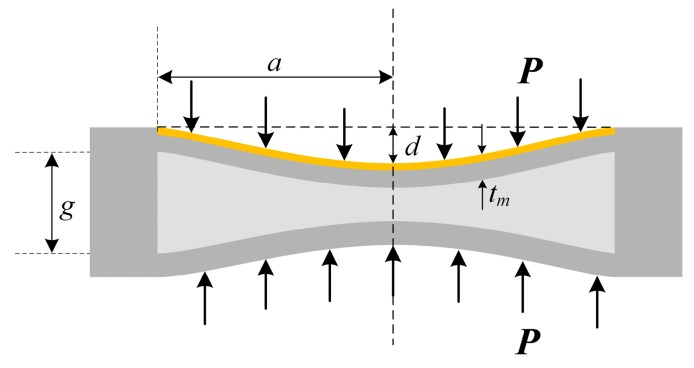
Deformation model of sensor cap with loaded pressure.

**Figure 4:**
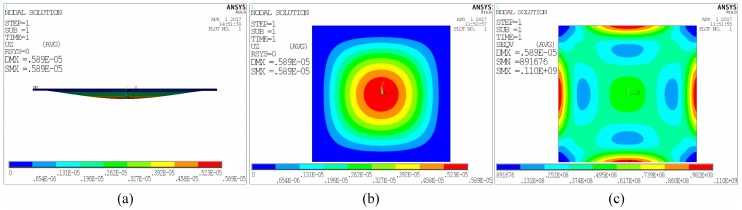
ANSYS simulation of the plate under the uniform load of 120 kPa barometric pressure. (**a**,**b**) Deflection distribution of the pressure-deformable plate; (**c**) Von Mises Stress distribution.

For the sensor proposed in this work, the pressure-sensitive cap is a rectangular mechanical model as shown in Figure 3, where *t_m_* is the thickness of the deformed plate and *d* is the maximum central deflection. Since the four edges of the plate are clamped and the length of each side meets the condition *d* << *t_m_* according to [16] (where *a* donates half of the length of the plate side), the model can be characterized with the small deflection bending theory of thin plate (*t_m_* << 2*a*). The maximum central deflection of a single plate can be expressed as [16,17]

(8) $$d=0.00126\frac{Pa^{4}}{D}$$

where *P* is the loaded pressure, and *D* is the flexural rigidity of the material which can be expressed by

(9) $$D=\frac{Et_{m}{}^{3}}{12(1-\nu^{{}^{2}})}$$

where *E* is the Young’s modulus and *v* is the Poisson’s ratio of the deformed material. It can be concluded from the ANSYS simulation results that when a single plate is uniformly loaded with 120 kPa barometric pressure, the maximum central deflection is 5.89 μm as shown in Figure 4a,b, and the maximum stress is 110 MPa which is distributed along the four edges, as represented by the red section in Figure 4c. Since the maximum stress is lower than the bending strength of 320 MPa, plastic deformation and rupture are not caused.

**Figure 5:**
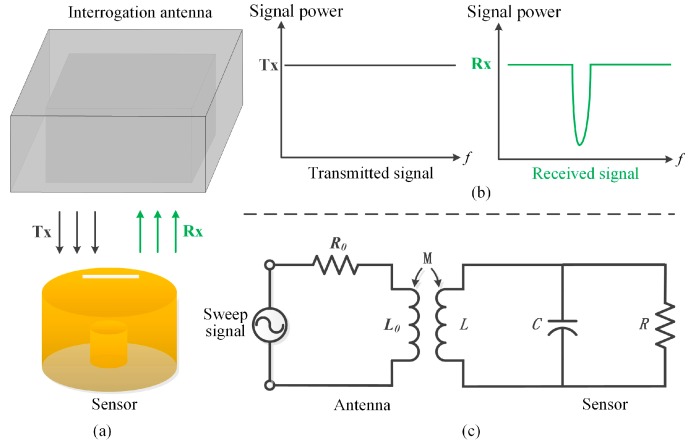
Wireless sensing mechanism of the pressure sensor.

To realize wireless signal transmission between the sensor and the readout device, a standard waveguide antenna is used, which acts as a virtual waveguide-coaxial transformer that can transmit a series of sweep interrogation signals, containing the sensor resonant frequency. To interrogate the sensor, a slot was cut off on the cap, allowing the linearly polarized electromagnetic wave transmitted from the interrogation antenna to couple with the re-entrant resonator, namely, the metal-coated cap where a slot acts as a response antenna. This has been represented in Figure 5a. Because of the presence of an open slot, the re-entrant resonator resonant frequency and the electromagnetic field distribution display a small variation in comparison with the resonator that is entirely metal-enclosed. We can verify this by comparing the results of the HFSS simulation with the sensor model constructed under eigenmode and waveguide drive mode, respectively. However, for the resonator model equipped with a slot, this is not a major concern and it can still work properly in accordance with the field distribution and resonant frequency of the enclosed resonator. For the received electromagnetic wave having multiple frequency components, only the specific wave whose electromagnetic field component is in agreement with the re-entrant resonator dominant mode, could generate continuous oscillations of standing-wave inside the resonator. The waves corresponding to other frequency components are reflected from the slot antenna and return to the interrogation antenna. This demonstrates the signal transmission process of the backscattering pressure sensor as shown in Figure 5b. By extracting the value of return loss (S11) of the interrogation antenna via a vector network analyzer (VNA), the resonant frequency of the re-entrant resonator can be obtained, which is accompanied by a negative peak. Figure 5c represents the equivalent lumped circuit model corresponding to the signal coupling between the interrogation antenna and the sensor. Since we know its working principle, the coupling phenomenon is clearly visible to us. *L*_0_ and *R*_0_ are the equivalent inductance and resistance of the interrogation antenna and *M* is the mutual inductance between the interrogation antenna and the sensor.

**Figure 6:**
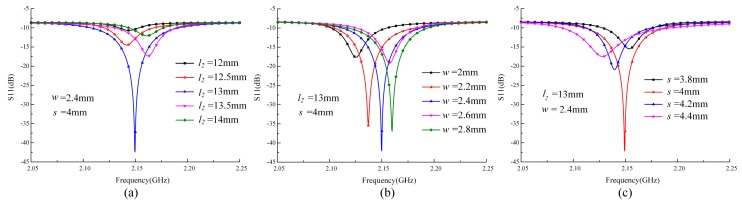
Design of the slot antenna in HFSS. Parameters simulation of (**a**) aperture length *l_2_*; (**b**) aperture width *w* and (**c**) aperture location *s*.

When pressure is loaded on the plates of the sensor cap, the resonant frequency of the re-entrant resonator changes due to the reduction of the gap *h*. Hence, the measurement of pressure can be realized by evaluating the relationship between resonant frequency and pressure. The wireless passive signal readout method will cause severe attenuation of the signal energy, mainly due to the path-loss. Hence, here we focus on the impedance matching between the re-entrant resonator and slot antenna because good impedance matching leads to higher sensor resolution and longer distances over which wireless sensing may be conducted. To enhance the signal coupling between the slot antenna and the re-entrant resonator, Ansoft HFSS software is used to design and optimize the dimensions and positions of the slot by utilizing the model illustrated in Figure 1. Since impedance matching is related to the return loss (S11), we can obtain the optimized dimensions of the slot antenna by comparing the values of S11 obtained from the simulated curve. The HFSS simulation results of a slot antenna having a width *w*, length *l*_2_, and location dimension *s* are shown in Figure 6. With the incorporation of several variables, a good matching is obtained along with a relatively small return loss.

The final dimensions of the sensor model are listed in Table 1.

## 3. Fabrication of the Pressure Sensor and Method of Pressure Sensor Measurement

**Figure 7:**
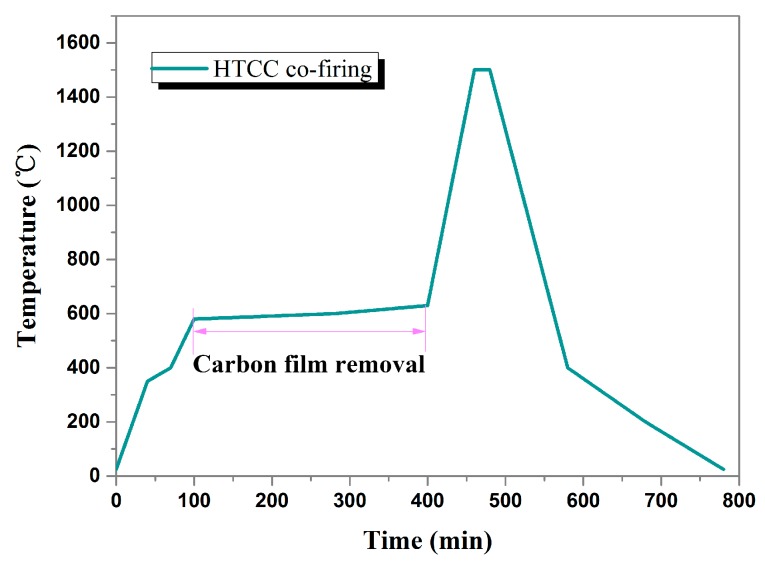
HTCC prepared alumina high-temperature sintering curve of the sensor cap.

**Figure 8:**
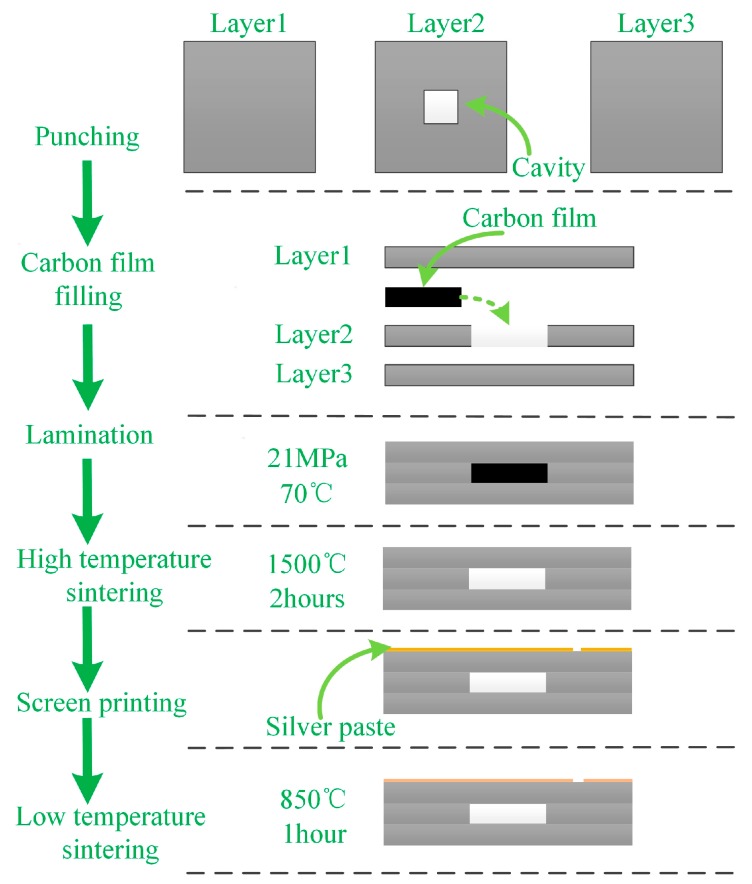
Fabrication process of sensor cap with air-cavity.

Owing to the rapid development of micro-machining and HTCC technologies, the proposed pressure sensor with optimized dimensions can be fabricated with ease and precision. The sensor substrate is fabricated with alumina ceramic by adding an internal post to form a cylinder and the structure of an inner torus channel. Next, the ceramic surface is polished to satisfy the requirements of roughness and dimension. The sensor cap with a sealed air-cavity is fabricated using the HTCC technology [8]. The ESL 44000 (ESL ElectroScience, King of Prussia, PA, USA) green tape was used as the HTCC material and its properties are listed in Table 2. The main fabrication process is roughly composed of drilling, carbon film filling, lamination, high-temperature sintering (sintering curve is plotted in Figure 7), screen-printing, and low temperature sintering. These steps have been illustrated in Figure 8. First, a cavity hole was drilled, which was subsequently filled with carbon film to support the pressure-sensitive plates and prevent them from collapsing and cracking during the lamination process. Afterwards, the laminated sensor cap was sintered in a muffle furnace. 

**Figure 9:**
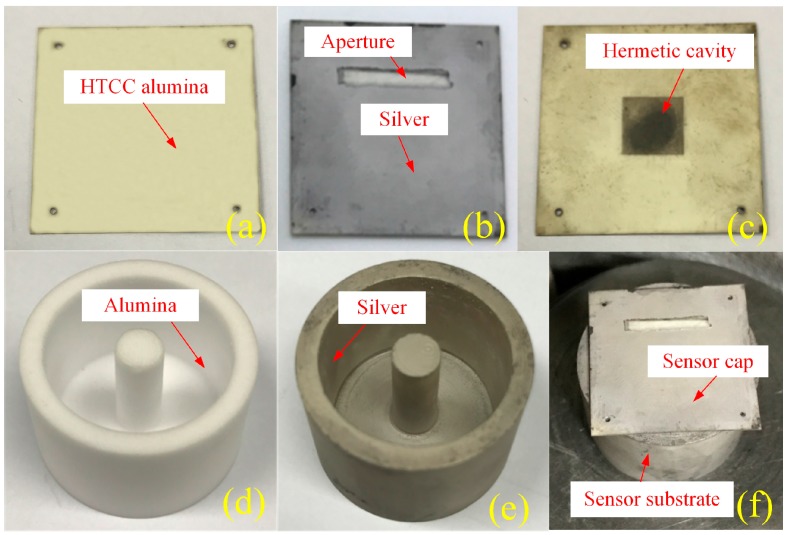
Fabrication processes of the sensor. (**a**) Sensor cap after high-temperature sintering; (**b**) Silver-coated slot antenna on the sensor cap; (**c**) Back side of the sensor cap; (**d**) Sensor substrate made in alumina ceramic; (**e**) Silver-coated sensor substrate; (**f**) Assembled sensor.

It should be noted that the green tapes before sintering are porous with various little holes. This could be known from the ESL44000 data sheet. The ESL44000 is a flexible cast film of 96% alumina powder dispersed in an organic matrix. This material gets denser during sintering from 25 °C to 1500 °C to yield a hard and white-colored alumina ceramic that is not porous. During sintering, the carbon film in the cavity reacts with the air which enters via the little holes and eventually turns into CO_2_ exhausting via the holes. An air-sealed cavity is formed at the end of the sintering process. And the pressure inside the cavity is estimated to be one bar, owing to the final equilibration between the inner and external pressure. After high temperature sintering of the sensor cap, the slot antenna patterns were printed on its surface by the screen-printing technique, for which Dupont 6142D silver (Dupont, Wilmington, NC, USA) paste was used. Next, it was placed inside a muffle furnace at 125 °C for 15 min to dry the silver paste followed by sintering at a peak temperature of 850 °C for 1 h. Finally, the silver-coated substrate and the sensor cap were aligned, assembled, and fixed by evenly coating a layer of high temperature resistant glue between the contact surface of sensor cap and substrate. The cap is glued on the substrate but not hermetically because we didn’t coat the glue for the whole contact surface between the cap and the substrate, while just coat for several contact points. The as-fabricated silver-coated sensor cap and sensor substrate are shown in Figure 9a–e respectively; the assembled sensor is shown in Figure 9f. It should be noted that to eliminate the influence of skin effect, the thickness of the printed metal should be ten-fold of the skin depth [18]. For silver, the skin depth can be calculated to be 1.26 μm corresponding to a frequency of 2.58 GHz. Hence, the thickness of the coated silver should be at least 12.6 μm.

**Figure 10:**
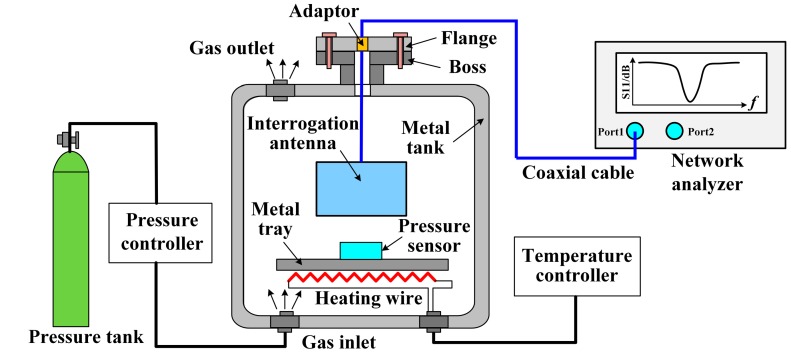
High-temperature pressure measurement platform.

For performing measurement with the fabricated pressure sensor, a complex platform composed of a gas tank, a sealed metal chamber and a VNA has been developed for the precise control of temperature and pressure. Figure 10 shows the schematic of the sensor’s measuring platform developed for this work. A waveguide of coaxial adapter (right angle) was used as the interrogation antenna owing to its relatively wide bandwidth and small size. This could be inserted into the chamber, whose end was connected to the Agilent network analyzer E5061B (Agilent Technologies, Santa Clara, CA, USA) by a coaxial cable and an adaptor. The sensor was placed on the metal tray with a wireless reading distance of 10 mm.

**Figure 11:**
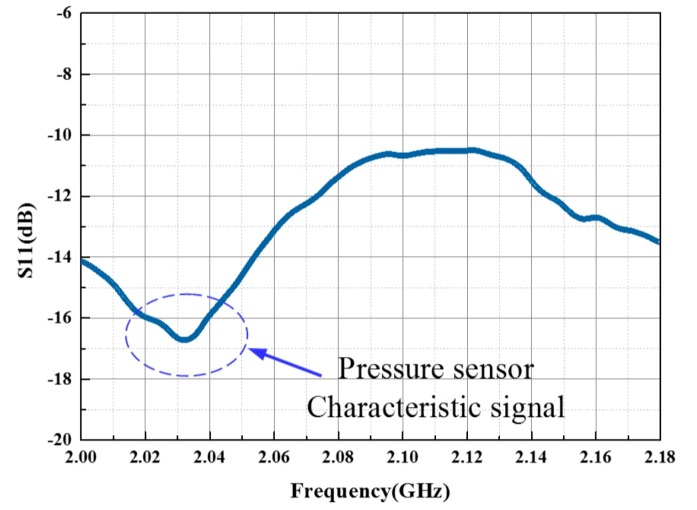
Original sensor response in metal sealed chamber.

While operating, the sensor could work at the resonant frequency of 2.1065 GHz in an open environment which has a deviation from the calculated resonant frequency of 2.58 GHz. The possible reasons could be the existence of modeling errors in the calculation of equivalent values of *L* and *C* and the assumption of alumina being a non-magnetic material. It was found in Figure 11 that the readout signal with a relatively gentle negative peak of the unloaded sensor may be overlooked during detection. If we load gas pressure on the sensor at higher temperatures, it is possible to lose the characteristic frequency.

**Figure 12:**
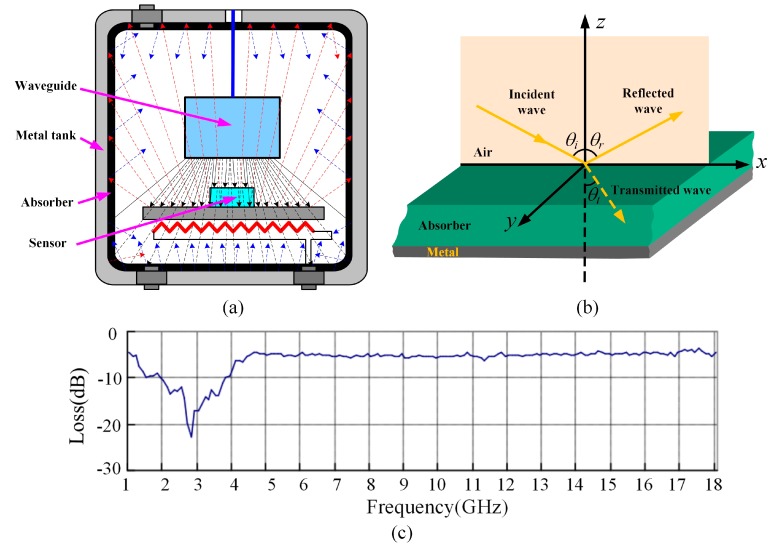
(**a**) Schematic of the measured pressure sensor in the metal sealed chamber; (**b**) Operating mechanism of electromagnetic wave absorber with incident angle *θ_i_*, reflected angle *θ_r_*, and transmitted angle *θ_t_*; (**c**) RATG-2 GHz-3 mm absorber performance curve.

As depicted in Figure 12a, the interrogation electromagnetic wave is strongly reflected by the metal chamber wall, and could even overshadow the sensor response signal. In order to solve this problem, microwave absorbing material was pasted on the inner wall of the metal sealed chamber to provide electromagnetic shielding for the interrogation antenna and the pressure sensor. The working mechanism of the electromagnetic wave absorber is shown in Figure 12b. By converting the absorbed electromagnetic waves into heat or other forms of energy, the incident electromagnetic wave is effectively absorbed. Impedance matching and attenuation are the two main factors for evaluating the performance of the absorber. Generally, the reflectance *R* < −10 dB is used to evaluate the absorption properties of the wave absorber. For performing reflectivity measurements of the electromagnetic wave absorber, the length of the side of the absorber should be larger than 5*λ* (*λ* is the wavelength of the incident wave). This reduces the influence of edge diffraction. In this experiment, with the measurement frequency being in the range of 1–3 GHz, a square of size 200 mm × 200 mm, having a thickness of 3 mm was adopted to meet the requirements of the dimensions. Here, a rubber absorbing material of type RATG-2 GHz-3 mm is chosen due to its good microwave absorbing characteristic. Furthermore, for a perpendicularly incident electromagnetic wave, it has a reflectance *R* < −10 dB with a relative bandwidth of 35%, and is temperature resistant up to 250 °C. The absorber performance curve of RATG-2 GHz-3 mm is shown in Figure 12c. For the sensor designed in this work, with a resonant frequency of 2.1065 GHz, the reflectance *R* is about −12 dB which meets the requirement of it being less than −10 dB.

**Figure 13:**
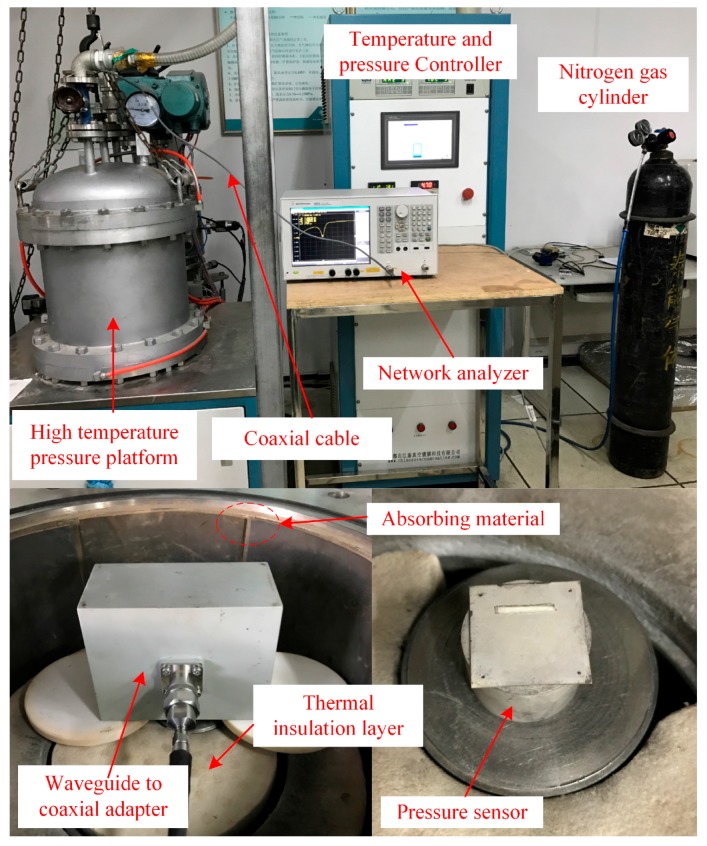
High-temperature pressure measurement platform developed for the fabricated sensor.

**Figure 14:**
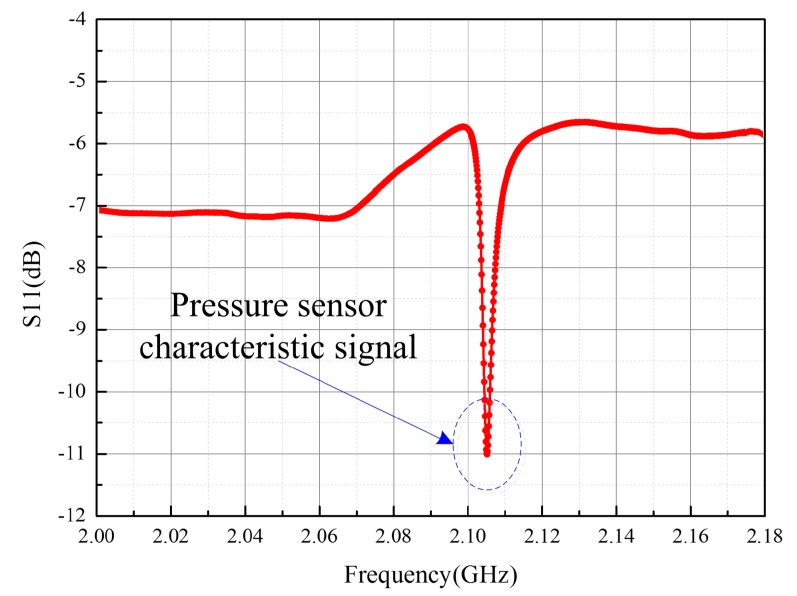
Pressure sensor response signal detected with electromagnetic wave absorbing material pasted on the inner wall of the metal chamber.

The developed high-temperature pressure measurement platform equipped with an electromagnetic wave absorbing material is shown in Figure 13. Since the limit of temperature tolerance of the electromagnetic wave absorbing material is 250 °C and the temperature resistance of the coaxial cable is low, a thermal insulation layer was utilized. Consequently, the response curve, shown in Figure 14, becomes more distinguishable, which verifies the feasibility of the aforementioned electromagnetic shielding method.

**Figure 15:**
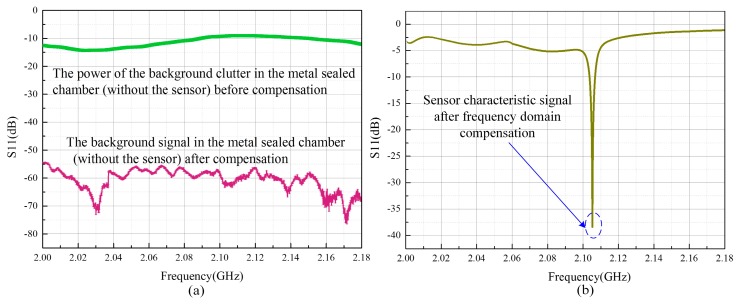
(**a**) Signal of the background clutter before and after frequency-domain compensation in the metal-sealed chamber; (**b**) Sensor characteristic signal after compensation in the frequency domain.

To raise the signal to noise ratio (SNR) of the readout signal further, background compensation method is utilized in the frequency domain, as shown in Figure 15a. The upper curve represents the signal of the background clutter inside the metal-sealed chamber at a certain moment (in the absence of the sensor) before including the compensation step, while the lower curve represents the background signal taking into account the compensation in the frequency domain. The entire signal processing is achieved in the absence of the sensor via a VNA. Initially, the background clutter data is stored inside the memorizer of the network analyzer, following which the real-time background clutter data is used to subtract the afore-stored data in the frequency domain. From Figure 15a, a significant reduction in the average value of the background clutter, i.e., from −10 to −60 dB was observed, indicating that the frequency-domain background clutter compensation method could effectively improve the SNR of the sensor signal. Figure 15b depicts the measured sensor signal after the inclusion of compensation. It is observed that, in comparison with the curve shown in Figure 14, the one in Figure 15b is smoother and the characteristic signal of the sensor can be distinctly recognized.

**Figure 16:**
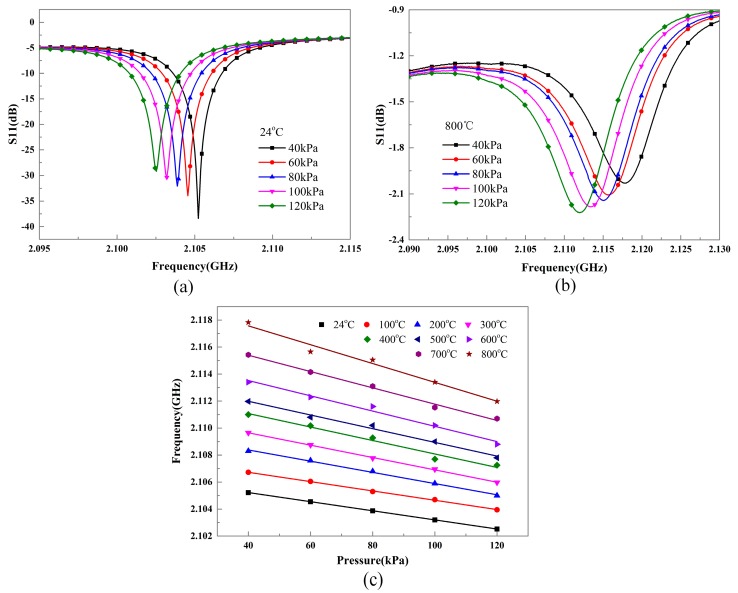
Pressure response curves of the sensor at temperature of (**a**) 24 °C and (**b**) 800 °C; (**c**) Sensor characteristic frequency curves of 40–120 kPa within 24–800 °C.

Next, for performing measurement, the pressure sensor was heated by the heating wire placed under the metal tray. The heating temperature was in the range of 24 °C to 800 °C, which could be incremented at a rate of 10 °C/min with a stabilization time of 20 min between each 100 °C mark. When the temperature value stabilized at separate points of 24 °C, 100 °C, 200 °C, 300 °C, 400 °C, 500 °C, 600 °C, 700 °C, and 800 °C, nitrogen was released into the metal-sealed chamber within a pressure range of 40 kPa and 120 kPa at an incremental step of 20 kPa. Figure 16a illustrates a group of pressure response curves of the sensor corresponding to 24 °C, where, with the increase of pressure, the characteristic frequency was found to drift towards lower frequency. The pressure response curves recorded at 800 °C, shown in Figure 16b, presented a similar tendency of frequency variation, but the signal strength was obviously attenuated. The attenuation could be due to the increased dielectric loss (for the increase of the loss tangent) of the alumina ceramic and metallic loss (for the increase of equivalent resistance) of the silver layer under the high temperature environment, thus resulting in a sharp fall of the *Q* factor.

The variation of the whole group of sensors’ characteristic frequencies as a function of pressure at different temperatures are subsequently extracted and the corresponding linearly fitted curves are shown in Figure 16c. It can be concluded that for a constant temperature, the relationship between the sensor’s characteristic frequency and pressure is approximately linear, and with the increase of the pressure, the characteristic frequency decreases. The maximum value of the decrease in the characteristic frequency in the range 40–120 kPa is 5.85 MHz at 800 °C. From this, the value of maximum sensitivity can be obtained as 73.125 kHz/kPa. The sensitivity shows a tendency to increase in the range of 24 °C to 800 °C. This is due to the reduction of the Young’s modulus of alumina ceramic at elevated temperatures, making the pressure-sensitive plates easily deformable. Thus, it shows a higher rate of decrease of the characteristic frequency. For the practical measurement of pressure, under conditions of high temperature, the temperature can be measured by the microwave-scattering principle as done in a previous report [9]. In the case of high temperature-resistant HTCC material, higher values of temperature can be measured. 

However, the drift in temperature at a constant value of pressure cannot be ignored. The characteristic frequency of the sensor is found to increase at elevated temperatures in the range between 24 °C and 800 °C. The maximum value of the increase in the characteristic frequency is 12.6 MHz under the pressure 40 kPa from 24 °C to 800 °C. We conclude that the increasing values of permittivity of alumina ceramic dielectric at elevated temperatures would cause the characteristic frequency to decrease to a certain value, but the expansion of the air trapped inside the HTCC prepared alumina cavity would lead to an increase in the distance between the plates, resulting in a further increase in the frequency.

Furthermore, in a practical scenario, there are low chances to find applications with wall-covered enclosure and it’s costly to prepare such a measurement environment as we have set. Hence, a planar sensor that can fully make use of the electromagnetic wave reflection property of the metal wall would be better fit the practical harsh environment. We will carry out further study on this subject for its application in more scenarios. On the other hand, the background compensation method using VNA is inconvenient in practical scenarios due to its high cost and big shape. A portable device would be better for the measurement of the wireless sensor. We tend to extract one of the modules from VNA for measurement of S parameters. Such equipment needs to be customized with limited measurement functions but with a low profile.

## 4. Conclusions

This paper presents the results of the design, fabrication, and testing of a pressure-modulating re-entrant resonator sensor. For the design of the sensor, we modified the theoretical calculation of the model. HTCC technology was applied to fabricate the sensor cap with a sealed cavity, whose advantage is that it does not require any extra sealing between the cap and the substrate. The method of sensor signal extraction by frequency-domain compensation was used to suppress the background noise. To reduce the reflection of electromagnetic wave in the metal-sealed environment, an electromagnetic wave absorber was applied. The sensor was used to measure pressure, with a working range of 40 kPa to 120 kPa, corresponding to temperatures ranging from 24 °C to 800 °C. The values of maximum pressure sensitivity and maximum temperature drift were 73.125 kHz/kPa and 15 kHz/°C, respectively. The proposed pressure sensor shows great potential for application in harsh environments and our future work will focus on improving the gain and directivity of the interrogation antenna to increase the sensing distance. We will also focus towards reducing the dimensions of the sensor and replacing of silver paste with platinum paste to gain higher operation temperatures up to 1500 °C. An integrated sensor for simultaneous measurement of temperature and pressure will be developed to calibrate the frequency shift of the pressure sensor under high temperature conditions.

## Figures and Tables

**Table 1:** Dimensions of the re-entrant resonator sensor model.

Symbol	Value (mm)	Symbol	Value (mm)
*h*	0.7	*w*	2.4
*h*_1_	15.3	*l*_1_	25
*h*_2_	16	l_2_	13
*r*_1_	3	*s*	4
*r*_2_	11	*a*	3
*g*	0.1		

**Table 2:** Properties of the ESL 44000 green tape.

Property	HTCC/ESL44000 Alumina
*ε_r_*	9
tanδ	0.0001
E/(GPa)	380
Poisson’s Ratio	0.24
Green Tape thickness/(μm)	200 ± 20
X, Y shrinkage/(%)	16.0–18.0
Z shrinkage/(%)	15 ± 0.5
